# Fatal *Plasmodium falciparum*, *Clostridium perfringens*, and *Candida spp.* Coinfections in a Traveler to Haiti

**DOI:** 10.1155/2009/969070

**Published:** 2009-05-05

**Authors:** Gillian L. Genrich, Julu Bhatnagar, Christopher D. Paddock, Sherif R. Zaki

**Affiliations:** ^1^The George Washington University Medical Center, Washington DC 20037, USA; ^2^Infectious Diseases Pathology Branch, Division of Viral and Rickettsial Diseases, Centers for Disease Control and Prevention, 1600 Clifton Road, N.E., Mailstop G32, Atlanta, GA 30333, USA

## Abstract

Malaria is one of the most common causes of febrile illness in travelers. Coinfections with bacterial, viral, and fungal pathogens may not be suspected unless a patient fails to respond to malaria treatment. Using novel immunohistochemical and molecular techniques, *Plasmodium falciparum*, *Clostridium perfringens*, and *Candida spp.* coinfections were confirmed in a German traveler to Haiti. *Plasmodium falciparum*-induced ischemia may have increased this patient's susceptibility to *C. perfringens* and disseminated candidiasis leading to his death. When a patient presents with *P. falciparum* and shock and is unresponsive to malaria treatment, secondary infections should be suspected to initiate appropriate treatment.

## 1. Introduction

*Plasmodium falciparum*, an intraerythrocytic parasite that causes the most severe form of human malaria, is endemic to Haiti where it caused 32 739 infections and contributed to an estimated 741 malaria deaths in 2006 [1–3]. Malaria infection poses a risk to the 140 000 travelers from nonendemic countries that visit Haiti each year [4]. Progression to severe illness from initial symptoms of fever, headache, chills, and myalgia occurs rapidly through the phenomenon of sequestration, in which *P. falciparum*-infected erythrocytes attach to blood vessels and impede normal blood flow, particularly in the brain [5]. Poor perfusion leads to tissue ischemia, which may subsequently increase a patient's susceptibility to secondary infections [5, 6] such as *C. perfringens,* and *Candida spp*. *C. perfrignens* is an anaerobic spore-forming bacillus that produces a virulent hemolytic alpha toxin [7] and causes gas gangrene, a potentially deadly infection characterized by fever, pain, edema, myonecrosis, and gas production [8]. And in a setting of tissue ischemia and necrosis, endogenous gut microflora such as Candida may translocate across the epithelial border and gain access to systemic circulation resulting in disseminated candidiasis [9].

Malaria coinfections with *Leptospira spp*., *Coxiella burnetti*, *Brucella melitensis,* and *Streptococcus pneumoniae* as well as with enteric bacteria (*Eschericha coli* and *Salmonella*, and *Acinetobacter baumannii)* have previously been reported [10–14]. To our knowledge, this report describes the first fatal coinfection of *P. falciparum*, *C. perfringens*, and *Candida spp*.

## 2. Materials and Methods

### 2.1. Case Presentation

On October 29th 2005, a 56-year-old German tourist living in Haiti, with a history of heavy alcohol use and 2 malaria infections treated with chloroquine, developed symptoms of fever, headache, nausea, and diarrhea. On November 3rd he visited a local hospital in Côtes des Arcadins and was diagnosed with *P. falciparum* infection by peripheral blood smear. The patient was treated with chloroquine and doxycycline the same day but developed altered mental status and was hospitalized. Despite falling parasite counts, the patient's neurological condition continued to worsen and he was mechanically ventilated. Seizures, bloody stools, hematemesis, and decreased urinary output were also documented. Due to his worsening condition, he was transferred to a hospital in Miami the following day, November 4th. At the time of hospital admission in Miami, the patient had thrombocytopenia, diffuse intravascular coagulation, electrolyte imbalance, and acute renal failure. Blood smears showed few malaria parasites, but treatment with antimalarial agents was continued. Bronchoalveolar lavage was positive for *Klebsiella pneumoniae*, *Acinetobacter baumannii*, *Pseudomas aeruginosa*, and yeast forms.

Blood culture on November 4th was positive for *C. perfringens.* Vancomycin, amphotericin, piperacillin, phenytoin, lorazepam, and hydrocortisone were added to the treatment regimen. On November 5th blood culture revealed *Candida tropicalis*. An encephalitis panel was also negative. The patient continued to decline clinically and he expired on November 7th, 10 days after symptom onset.

### 2.2. Tissue Samples and Immunohistochemical Analyses

Formalin-fixed, paraffin-embedded tissue (FFPET) sections of trachea, lung, heart, liver, spleen, kidney, small intestine, and central nervous system (CNS) were submitted to the Infectious Diseases Pathology Branch (IDPB) at the Centers for Disease Control and Prevention for diagnostic consultation.

Tissue sections were evaluated by routine hematoxylin and eosin (H&E), GMS, gram and Steiner stains, and were subsequently tested by immunohistochemistry (IHC) for suspected pathogens using immunoalkaline phosphatase technique as described previously [15, 16]. In brief, FFPET sections were deparaffinized in xylene and rehydrated in a graded alcohol series. IHC pretreatment conditions varied by primary antibody, and as deemed appropriate, consisted of one of the following 2 methods. (1) Antigen retrieval was performed by incubating sections submerged in Antigen Retrieval Citra Solution (BioGenex, San Ramon, Calif, USA) in a steamer for 10 minutes or (2) sections were digested in 0.1 mg/mL proteinase K (Boehringer-Mannheim, Indianapolis, Ind, USA) solution for 15 minutes. Sections were then blocked with 20% normal sheep serum in Tris-saline-Triton (NSS/TST) and incubated with the primary antibody (see below) for 60 minutes on the autostainer (DAKO, Carpinteria, Calif, USA). Detection of the bound antibody was performed using a secondary biotinylated antimouse antibody (DAKO), alkaline phosphatase-conjugated streptavidin, and naphthol phosphatase-fast red chromogen reagent. Slides were rinsed, counterstained in Mayer's hematoxylin, and mounted with aqueous mounting medium.

The primary antibodies used in immunohistochemical assays included *P. falciparum* histidine rich protein-2 (HRP-2, Biodesign, Saco, Maine, dilution 1:1000), a protein only produced by *P. falciparum* [16]; a polyclonal antibody reactive with various *Clostridium* species including *C. perfringens*, *C. botulinum*, *C. sordelii*, *C. novyii*, and *C. subterminale* (Biodesign, dilution 1:1000) [15]; a polyclonal antibody reactive with *Candida* species (Meridian Life Sciences; dilution 1:200). Appropriate positive and negative controls were run in parallel.

### 2.3. Molecular Analyses

DNA was extracted from FFPET sections of small intestine using the QIAamp DNA minikit (Qiagen, Valencia, Calif, USA), following the tissue extraction protocol. A *C. perfringens-*specific PCR assay was performed using the High Fidelity PCR kit (Roche Diagnostics, Indianapolis, Ind, USA) according to the manufacturer's instructions to amplify 283-bp fragment of the phospholipase C (alpha toxin) gene. For further evaluation, two *P. falciparum-* specific PCR assays targeting the 18S rRNA gene were performed. Primers used in PCR assays were published previously [17, 18] and are described in Table 1. PCR assays were modified for the FFPET. For each primer set, annealing temperature was adjusted accordingly. PCR was carried out on a GeneAmp PCR System 9700 thermocycler (Perkin-Elmer).

Amplified PCR products were separated on 1.8% agarose gel, extracted from the gel by using QIAquick gel extraction kit (Qiagen), and cycle sequenced by CEQ 2000 dye terminator cycle sequencing with quick start kit (Beckman Coulter, Fullerton, Calif, USA) and the respective primers. Postreaction cleanup was done by centri-sep spin columns (Princeton Separations, Adelphia, NJ). The samples were sequenced on a CEQ 2000 XL sequencer (Beckman Coulter, Fullerton, Calif, USA). Search for homologies to known sequences was done using the nucleotide database of the Basic Local Alignment Search Tool (BLAST) at http://www.ncbi.nlm.nih.gov/BLAST.

## 3. Results

**Figure 1:**
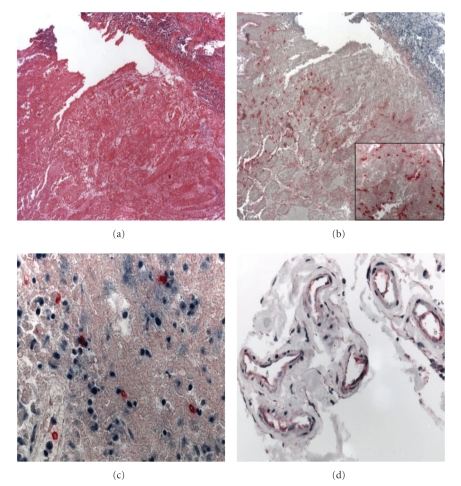
Histopathologic and immunohistochemical findings in representstive tissues of study patient. (a) Extensive necrosis of intestinal mucosa with diffuse, extensive, submucosal edema and multifocal inflammatory cell infiltrates (H&E, original magnification X13). (b) Abundant, diffuse immunostaining of *Clostridium spp*. antigens in intestine (original magnification X13; inset X100). (c) Immunohistochemical detection of *P. falciparum* HRP-2 antigens in pRBCs in spleen (original magnification X100). (d) HRP-2 antigen immunostaining in endothelium of CNS blood vessels (original magnification X50).

No hemozoin pigment was observed by routine hematoxylin-eosin (H&E) stain and no malaria parasites were seen in red blood cells on careful examination of multiple tissues. Histopathology of the colon and small intestine mucosa showed extensive necrosis with diffuse, extensive, submucosal edema and multifocal inflammatory cell infiltrates comprised predominately of neutrophils (Figure 1(a)). The serosa was moderately thickened and contained mixed inflammatory cell infiltrates. The liver showed autolysis with no significant inflammatory cell infiltrates. Spleen sections were congested and necrotic. The mucosal surface of the larynx was also extensively necrotic with abundant neutrophilic infiltrates. There were diffuse autolysis in the kidneys and intra-alveolar edema in the lungs. The heart showed interstitial edema. There were focal hemorrhages in the white matter of the cerebral cortex, but no conspicuous inflammatory cell infiltrates observed in the hippocampus, pons, cerebellum, and spinal cord.

The HRP-2 IHC assay revealed discrete immunostaining of intra-erythrocytic parasites in CNS, kidney, liver, heart, and spleen (Figure 1(c)). HRP-2 antigens were also detected in endothelium of systemic and CNS blood vessels (Figure 1(d)), and in renal tubular epithelium and renal casts. The *Clostridia spp.* IHC assay revealed abundant immunostaining in necrotic areas of the small intestine (Figure 1(b)); other tissues were negative. Small budding yeasts in the alveolar space of the lung were identified by an IHC stain for *Candida spp.*

Amplification products of expected sizes were generated by both the *C. perfringens* and *P. falciparum* PCR assays using DNA extracted from FFPET sections. Sequence analysis of positive amplicons also confirmed the infections of *P. falciparum* and *C. perfringens*.

## 4. Discussion

Immunohistochemical and molecular analysis of FFPET sections from the study patient confirmed coinfections with *P. falciparum*, *C. perfringens*, and *Candidia spp*. Malaria is one of the most common causes of fever in travelers [19] and nosocomial coinfections may occur with a frequency of 25%, according to a recent study of 96 fatal *P. falciparum* cases [20]. However, malaria coinfections are difficult to diagnose clinically and may only be suspected when a patient fails to respond to malaria treatment [10]. Several observations suggest that the patient described here, who presented to hospital with classic malaria symptoms, was successfully treated for malaria infection with chloroquine and doxycycline: (1) declining parasite counts on peripheral blood smear were documented during his hospital stay; (2) absence of hemozoin, a birefringent pigment produced by plasmodium in correlation with parasite density [21, 22], on H&E evaluation; (3) rare HRP-2 antigens detected by IHC in CNS, heart, lung, and liver sections as described in detail above. Rare staining was anticipated in this patient, considering that HRP-2 antigens are slow to clear from the blood and may persist in treated patients for up to two weeks [23]. The presence of amplification products of *P. falciparum* using PCR assays is not inconsistent with the H&E and immunohistochemical findings of resolving malaria infection. The primers used are highly sensitive, shown to detect as few as 0.7 parasites/mL [18]. In this context, and as our patient illustrates, PCR results must be correlated with the patient's clinical history.

The observation of necrosis in the small intestine supports the finding of *C. perfringens* in blood cultures found on November 4th, (day 7 of illness) after the patient was hospitalized in Miami. *C. perfringens* infection was subsequently confirmed by IHC testing and by PCR and sequencing analysis. *C. perfringens* is associated with several human diseases, including necrotizing enterocolitis [24], gas gangrene [25], antibiotic-associated diarrhea, and food poisoning outbreaks worldwide [26]. The alpha toxin is the most virulent of the 12 toxins produced by *C. perfringens* [9] because it destroys cell membranes, including those of red blood cells, platelets, and muscles. The bacterium also has sphingomyelinase activity that causes damage to the nerve-sheath in the central nervous system [8]. Deaths due to *C. perfringens* infections are rare in humans, and the portals of entry are usually surgical wounds [27]. However concomitant ischemia, or low oxygen tension in necrotic tissue, is a trigger for bacterial spore germination [28], and subsequent toxin production leads to anaerobic cellulitis or myonecrosis (gas gangrene) that rapidly progresses to severe sepsis [8]. The source of this patient's *C. perfringens* infection is unknown as *C. perfringens* is widespread in the environment and can be a component of normal human flora, but broad spectrum antibiotic use is suspected. It is likely that *P. falciparum* infection increased susceptibility to *C. perfringens* and *Candida* *spp*. in this patient.

*C. perfringens* infection causing intestinal myonecrosis may have begun with tissue ischemia due to *P. falciparum* sequestration, which is characterized by the attachment of parasitized erythrocytes (pRBCs) to endothelial cells lining blood vessels via a variety of constitutive receptors. Sequestration causes sluggish blood flow and disruption of microcirculation [6, 29] leading to ischemia. The infection subsequently leads to hyperlactemia, hypoglycemia, and metabolic acidosis, creating a dependence on anaerobic glycolysis for energy production [30]. Studies suggest that obstruction of the splanchnic blood vessels by pRBCs facilitates the entry of endotoxins and bacteria like *C. perfringens* from the digestive tract into the bloodstream [31]. While limited clinical data on this patient is available, this mechanism seems plausible and is supported by the tissue-based and molecular testing performed.

This patient's chronic alcohol consumption may also have contributed to the severity of multiple infections. Ethanol has been shown to decrease the respiratory burst activity of neutrophils [32], and heavy alcohol consumption (> or = 5 drinks per day) is significantly associated with ICU-acquired bacterial infection, even when controlling for duration of mechanical ventilation and other risk factors [33]. Further, the oral flora of heavy alcohol drinkers has been shown to differ significantly from the flora of nonalcoholics. One study showed that anaerobes, including *Clostridrium spp.*, are present in 84.5% of heavy drinkers, compared with 30.5% of nonalcoholics and similarly, *Candida spp.* were found in 34.5% of heavy drinkers whereas only 5.5% of nonalcoholics carried the microbiota [34]. The pathogens detected by bronchoalveolar lavage, *Klebsiella pneumoniae*, and *Acinetobacter baumannii* are particularly common causes of pneumonia in chronic alcoholics; while *Pseudomas aeruginosa* is associated with mechanical ventilation [35].

Disseminated candidiasis contributed to our patient's demise; *C. tropicalis* was detected by bronchoalveolar lavage, and our IHC analysis revealed *Candida* *spp.* in FFPET of small intestine. Animal studies have shown that mucosal damage and broad-spectrum antibiotics are important factors in opportunistic candidiasis [30]. In this case, we suggest that the use of broad spectrum antibiotic coverage in the setting of tissue ischemia and mucosal erosion secondary to *P. falciparum* and *C. perfringens* infections facilitated disseminated infection.

To our knowledge, this is the first report of *P. falciparum*, *C. perfringens*, and *Candida spp*. coinfections. In the case presented here, *P. falciparum* may have increased susceptibility to *C. perfringens* infection by inducing a state of hypoxia-ischemia. The patient's chronic alcohol consumption may have increased his susceptibility to intestinal necrosis and ischemia, which created a suitable environment for translocation and dissemination of *Candida spp.*, particularly in the setting of broad-spectrum antibiotic administration. *P. falciparum* remains a significant threat to travelers to Haiti and other areas where the parasite is endemic. This case highlights the importance of suspecting bacterial and fungal coinfections in patients refractory to malaria treatment.

## Figures and Tables

**Table 1:** Oligonucleotide primers used in the PCR assays.

Primer sequence (5′-3′)	Gene target	Product size (bp)	Anneling temperature (°C)	Reference
*Clostridium perfringens * specific PCR				

PL3 AAG TTA CCT TTG CTG CAT AAT CCC	Phospholipase C	283	55	Fach and Popoff 1997 [17]
PL7 ATA GAT ACT CCA TAT CAT CCT GCT	Phospholipase C			

*Plasmodium falciparum * specific PCR				

rFAL1 TTA AAC TGG TTT GGG AAA ACC AAA TAT ATT	18S rRNA	205	58	Perandin et al. 2004 [18]
rFAL2 ACA CAA TGA ACT CAA TCA TGA CTA CCC GTC	18S rRNA			
FAL-F CTT TTG AGA GGT TTT GTT ACT TTG AGT AA	18S rRNA	98	58	Perandin et al. 2004 [18]
FAL-R TAT TCC ATG CTG TAG TAT TCA AAC ACA A	18S rRNA			
